# Production, purification and biochemical characterisation of a novel lipase from a newly identified lipolytic bacterium *Staphylococcus caprae* NCU S6

**DOI:** 10.1080/14756366.2020.1861607

**Published:** 2020-12-17

**Authors:** Junxin Zhao, Maomao Ma, Zheling Zeng, Ping Yu, Deming Gong, Shuguang Deng

**Affiliations:** a State Key Laboratory of Food Science and Technology, Nanchang University, Nanchang, China; b Jiangxi Province Key Laboratory of Edible and Medicinal Resources Exploitation, Nanchang University, Nanchang, China; c School of Food Science and Technology, Nanchang University, Nanchang, China; d School of Resource and Environmental and Chemical Engineering, Nanchang University, Nanchang, China; e New Zealand Institute of Natural Medicine Research, Auckland, New Zealand; f School for Engineering of Matter, Transport and Energy, Arizona State University, Tempe, AZ, USA

**Keywords:** *Staphylococcus caprae*, lipase, purification, biochemical characterisation

## Abstract

A novel lipase, SCNL, was isolated from *Staphylococcus caprae* NCU S6 strain in the study. The lipase was purified to homogeneity with a yield of 6.13% and specific activity of 502.76 U/mg, and its molecular weight was determined to be approximately 87 kDa. SCNL maintained above 80% of its initial activity at a wide range of temperatures (20–50 °C) and pH values (6–11), with an optimal temperature at 40 °C and optimal pH at 9.0 with *p*-nitrophenyl palmitate as a substrate. SCNL exhibited a higher residual activity than the other staphylococcal lipases in the presence of common enzyme inhibitors and commercial detergents. The lipase activity was enhanced by organic solvents (isooctane, glycerol, DMSO and methanol) and metal ions (Na^+^, Ba^2+^, Ca^2+^, and Mn^2+^). The *K*m and *V*max values of SCNL were 0.695 mM and 262.66 s^−1 ^mM^−1^, respectively. The enzyme showed a preference for *p*-NP stearate, tributyrin and canola oil. These biochemical features of SCNL suggested that it may be an excellent novel lipase candidate for industrial and biotechnological applications.

## 1. Introduction

Lipolytic enzymes are composed of esterases (EC 3.1.1.1) and lipases (triacylglycerol hydrolases, EC 3.1.1.3), and the latter represent the most important enzymes that act on ester bonds between a carboxylic acid and an alcohol group at the lipid-water interface^1^. Therefore, they are widely applied to various areas of industries, including foods, pharmaceuticals, biodiesel, detergents, cosmetics and chemicals^2^. Lipases may come from a variety of sources, and are ubiquitous enzymes produced by some biological systems, such as plants, animals and microorganisms^3^. Furthermore, microbial lipases are a kind of crucial enzymes in biotransformation due to its multifunction in applications and convenience for mass production, and are superior to the lipases from plants and animals in terms of activity, stability, ease of purification, molecular modifications, and continuous production independent of season^4^. The special properties of these enzymes were given by the environments where microorganisms were isolated occasionally^5^.

Lipolytic bacteria are classified into different families based on their gene sequences and biochemical properties, and a high level of lipase is also a general feature of staphylococcus. The lipolytic enzymes in staphylococcus that have been studied for their applications in biotechnology^2^ are mainly extracellular lipases and expressed as prepropeptides, and several staphylococcal lipases were purified and biochemically characterised, with the molecular weight of approximately 70 kDa^6^. In recent years, our major research direction focussed on lipases from staphylococcal species, and to our knowledge no *Staphylococcus caprae* lipases have been reported, and thus purification and characterisation of these enzymes are necessary.

In this study, we screened and identified a new lipolytic bacterium *Staphylococcus caprae* NCU S6 from sewerage, evaluated the growth characteristics and optimised the culture conditions of the strain for a maximum production of a novel lipase. The obtained lipase was purified and biochemically characterised.

## 2. Materials and methods

### 2.1. Materials

All chemicals were purchased from Aladdin Chemical Co. (Shanghai, China) and Sigma–Aldrich (St. Louis, MO, USA). Sephadex G-50 and DEAE-Sepharose Fast Flow column were provided by Beijing Solarbio Science & Technology Co. *Staphylococcus epidermidis* ATCC 12228 and *Staphylococcus aureus* ATCC 25923 were obtained from Guangdong Huankai Microbial Science & Technology Co. (Guangzhou, China). All chemicals used in this study were of analytical grade.

### 2.2. Isolation and molecular identification of lipase-producing strains

The samples from oil contaminated sewerages at Nanchang University were enriched in the 25 ml lysogeny broth (LB) medium, pH 7.0 with shaking at 150 rpm. Initial lipolytic bacteria were screened by using bromocresol purple (0.2%, *w/v*) and tributyrin (1.0%, *v/v*) agar plates^7^. Positive isolates were rescreened by using Rhodamine B (0.5%, *w/v*) agar plate for lipase production^8^.

The bacteria were characterised by morphological, biochemical and molecular techniques. The biochemical tests, including coagulase test, novobiocin (5 μg/piece) sensitivity test and catalase production test, were carried out to discriminate staphylococci^9^, and *Staphylococcus aureus* ATCC 25923 and *Staphylococcus epidermidis* ATCC 12228 were used as standard strains. The isolate was further identified by the 16S rRNA gene sequencing method. Then the genomic DNA of NCU (Nanchang University) S6 was obtained from colonies by protease K cleavage method and amplified as described previously^10^. Evolutionary relationship was determined by MEGA version 7.0.

### 2.3. Culture conditions

The *S. caprae* strain was cultured overnight at 37 °C and 200 rpm in 250 ml Erlenmeyer flasks with 50 ml medium A containing 1% (*w/v*) glucose, 0.5% yeast extract, 1% tryptone and 0.5% NaCl, pH 7.0. After preincubation, 4% (*v/v*) medium A was inoculated into 50 ml medium B (fermentation medium, pH 7.0): 0.5% (*w/v*) glucose, 2.5% tryptic soy broth, 0.25% K_2_HPO_4_, 1% NaCl, 2% (*v/v)* olive oil and 1% Tween-80. Then 51 h-incubation was carried out in an orbital shaker at 37 °C and 200 rpm.

### 2.4. Measurement of lipase activity

The lipase hydrolysis activity towards *p*-nitrophenyl palmitate (NPP) was measured according to the colorimetric method^11^, with some modifications. Briefly, a reaction mixture contained 100 μL *p*-NPP (7.5 mM, methanol solution) and 2.1 ml buffer A (50 mM Tris-HCl, pH 8.0). After heating of the samples in a water bath for 10 min at 40 °C, 100 μL appropriately diluted enzyme was added. The reaction mixture was then incubated at 40 °C for 20 min with a control group using thermally inactivated lipase to consider any spontaneous hydrolysis of *p*-NPP. The reaction was terminated by adding 100 μL ZnSO_4_ (100 mM, sterile water) on ice bath. Reaction solution was centrifuged at 8,000 × g for 5 min to remove the insoluble substances, and the supernatant was used to determine the amount of liberated *p*-nitrophenol (NP) by its absorbance at 410 nm. The lipolytic activities were expressed as international units. One unit of enzyme activity corresponded to one micromole of *p*-NP released from *p*-NPP per minute, and the specific activities were expressed as U/mg of protein.

### 2.5. Measurement of protein concentration

Protein concentration was determined according to Bradford^12^ by using bovine serum albumin (BSA) as a standard. The supernatant obtained from submerged culture medium after centrifugation at 4000 × g for 30 min was used as crude extract for subsequent purification steps.

### 2.6. Evaluation of growth characteristics

After culturing the purified single strain in medium A (50 ml) overnight, the inoculum at 8% (*v/v*) was transferred into medium B (50 ml). The 5 days-incubation was carried out in an orbital shaker at 37 °C and 200 rpm. Then, the absorption value of fermentation medium at 600 nm was measured every 3 h by using TU-1950 Double-Beam UV-Vis spectrophotometer (Beijing Purkinje General Instrument Co., Beijing, China). The growth characteristics of the strain were recorded as time-OD value curve. Meanwhile, the fermentation medium was taken out every 3 h and centrifuged at 8000 × g for 15 min to get the supernatant for lipase activity measurement.

### 2.7. Lipase production and purification

The *S. caprae* NCU S6 was cultivated under optimal reaction conditions for 51 h at 37 °C and 200 rpm. The submerged culture medium was centrifuged at 4000 × g for 30 min at 4 °C to discard the cells. The clear supernatant containing extracellular lipase was used as crude lipase for subsequent purification. The lipase produced by *S. caprae* was purified by three steps and the purified lipase named as SCNL. The lipase activity and protein concentration were measured after each step.

#### 2.7.1. Ammonium sulphate precipitation (ASP), ultrafiltration and lyophilisation

Gradient concentration of ammonium sulphate (10 − 80%) was added to cell-free crude extract under magnetic stirrer at 4 °C for overnight stratification. The precipitate at 80% saturation ammonium sulphate solution was obtained by centrifugation at 8000 × g for 30 min and resuspended in Buffer A. The crude lipase solution was centrifuged at 8000 × g for 5 min to remove the insoluble materials and then concentrated by ultrafiltration with Amicon-Ultra-15 (MWCO10kD, Millipore, Bedford, MO, USA). The concentrated lipase was lyophilised by a freeze drier after dialysing for follow-up experiments.

#### 2.7.2. Gel filtration chromatography (GFC)

The freeze-dried powder of enzyme solution (10 ml, Buffer A) was loaded onto a Sephadex G-50 column (16 × 300 mm) previously equilibrated with Buffer A at a flow rate of 50 ml/h for 1 h. The fractions showing the highest lipase activity were collected.

#### 2.7.3. Ion-exchange chromatography (IEC)

The enzymatically active fractions (40 ml) eluted from the Sephadex G-50 column were loaded onto a DEAE-Sepharose Fast Flow column (16 × 300 mm) preequilibrated with Buffer A. The lipase was absorbed by the column, and the impurities were washed away by Buffer A at a flow rate of 50 ml/h until no lipase activity was detected in the washed fractions. Absorbed proteins were subjected to gradient elution, followed by a series of NaCl solutions (100 ml of 0.02–1.0 M in sterile water) at a flow rate of 20 ml/h. The fractions with the highest lipase activities were concentrated by ultrafiltration with Amicon-Ultra-15 (MWCO10kD, Millipore, Bedford, MO, USA), and then freeze-dried for subsequent analyses.

### 2.8. Gel electrophoresis

Analytical polyacrylamide gel electrophoresis of SCNL was carried out in sodium dodecyl sulphate (SDS-PAGE) following a previous method^13^.

### 2.9. Biochemical characterisation

#### 2.9.1. Effect of temperature and pH on lipase activity and stability

To determine the optimal reaction temperature of SCNL, SCNL was incubated in Buffer A at the temperatures from 10 to 60 °C, with a constant pH value at 7.0. The relative lipase activity and specific activity were determined under standard assay conditions. The highest lipase activity was taken as 100%. For thermostability determination, the purified lipase was pre-incubated in Buffer A at the same temperature range for 240 min. The mixture was sampled every 20 min and remaining lipase activity was determined as described previously. The initial lipase activity was taken as 100%.

The optimal pH value for the lipase activity was also investigated by assaying the activity in various buffers at different pH values (3.0–12.0) for 30 min at 40 °C. Sodium acetate (50 mM, pH 3.0–5.0), sodium phosphate monobasic (50 mM, pH 6.0–7.0), Tris-HCl buffer (50 mM, 8.0–9.0) and glycine/NaOH buffer (50 mM, 10.0–12.0) were used as buffers. For pH stability test, the purified lipase was pre-incubated in different buffers with pH 3.0 to 12.0 for 7.0 h. Then, the remaining activity was determined every hour as described previously.

#### 2.9.2. Effects of organic solvents on the stability of the lipase

The effect of organic solvents on the stability of the lipase was investigated by mixing different organic solvents (Table 1) with the enzyme in Buffer A at 10, 30, or 50% (*v/v*) for 1 and 28 days. The mixture was incubated at 37 °C and 200 rpm, and the control group (without organic solvents) was also prepared in Buffer A. The residual activity of the lipase was measured under standard conditions, samples were withdrawn periodically and the final concentrations of organic solvents were less than 3% (*v/v*).

**Table 1.** Effect of organic solvents on lipase activity.

Solvents	Concentration (%, *v/v*)	Residual activity (%) 1 day	28 days
Control	0	100.0 ± 1.1	100.0 ± 1.3
Acetone	10	96.9 ± 3.3	89.6 ± 2.1
	30	85.4 ± 1.1	77.9 ± 1.4
	50	59.2 ± 2.5	50.3 ± 1.0
Ethyl acetate	10	105.8 ± 1.9	80.4 ± 2.3
	30	95.1 ± 3.4	71.9 ± 1.7
	50	76.4 ± 3.7	64.5 ± 0.5
Isooctane	10	106.6 ± 1.2	110.6 ± 0.9
	30	109.0 ± 2.2	119.3 ± 0.2
	50	123.7 ± 3.2	124.9 ± 1.5
Diethyl ether	10	105.0 ± 0.9	93.7 ± 1.7
	30	90.8 ± 1.0	84.6 ± 3.2
	50	68.4 ± 2.5	75.9 ± 1.2
Glycerol	10	105.9 ± 0.8	114.9 ± 1.1
	30	115.0 ± 2.8	120.6 ± 0.2
	50	133.0 ± 1.4	136.4 ± 1.3
Ethylene glycol	10	105.0 ± 2.1	94.3 ± 0.6
	30	81.8 ± 1.8	75.1 ± 1.1
	50	58.2 ± 0.2	60.3 ± 1.4
	10	106.6 ± 4.7	100.7 ± 1.6
Petroleum ether	30	98.3 ± 1.7	89.1 ± 0.8
	50	81.4 ± 0.7	73.6 ± 0.5
	10	105.8 ± 1.0	86.2 ± 1.1
Acetonitrile	30	85.1 ± 3.4	72.3 ± 1.2
	50	76.4 ± 0.5	60.1 ± 1.5
	10	113.7 ± 1.6	105.6 ± 0.2
N-hexane	30	98.3 ± 1.3	90.5 ± 1.7
	50	78.2 ± 0.9	69.5 ± 3.1
	10	120.4 ± 1.4	109.5 ± 1.0
N-heptane	30	105.7 ± 0.9	95.2 ± 2.5
	50	98.2 ± 0.4	86.4 ± 1.8
	10	114.1 ± 0.7	110.1 ± 0.9
Ethanol	30	94.7 ± 2.3	91.2 ± 1.3
	50	84.8 ± 1.9	79.6 ± 2.1
	10	106.3 ± 1.7	108.3 ± 0.6
DMSO	30	100.1 ± 1.5	97.7 ± 1.4
	50	90.2 ± 3.3	91.2 ± 2.2
	10	116.4 ± 1.2	108.3 ± 2.8
Methanol	30	95.6 ± 1.8	98.1 ± 3.5
	50	71.9 ± 4.5	80.4 ± 1.9
	10	104.1 ± 0.7	105.7 ± 1.5
Isopropanol	30	94.7 ± 2.3	90.1 ± 1.2
	50	69.2 ± 1.1	76.4 ± 0.9

Enzyme samples were mixed with organic solvents (10, 30, 50%, *v/v*) and incubated for 1 day or 28 days in a rotary shaker set at 200 rpm and 37 °C prior to determining the residual activity. Results are presented as means ± standard deviation (*n* = 3).

#### 2.9.3. Effects of metal ions, inhibitors and detergents on the stability of the lipase

The effects of metal ions, inhibitors and detergents on the stability of the purified lipase activity were investigated. The relative activity was measured by pre-incubating the lipase solution with each additive. All reagents were prepared in Buffer A, with each metal ion at a final concentration of 1 or 10 mM (Table 2). Reaction mixtures containing 0.1 or 1% (*v/v* or *w/v*) of popular commercial detergents and inhibitors (Table 3) were also incubated at room temperature for 30 min. The lipase activity without addition of metal ions, inhibitors or detergents was defined as 100%, and the relative activity was determined as compared to the control. Experiments were performed as described above, using *p*-NPP as a substrate. Each assay was carried out in triplicates.

**Table 2.** Effects of some metal ions on the lipase activity.

	Relative activity (%)	
	1 mM	10 mM
Control	100.0 ± 1.8	100.0 ± 1.1
Metal ions		
Ag^+^	96.2 ± 1.5	101.7 ± 0.9
Na^+^	100.7 ± 2.6	120.8 ± 1.1
K^+^	95.8 ± 3.3	102.3 ± 1.2
Sn^2+^	13.4 ± 0.8	12.1 ± 0.5
Ba^2+^	102.6 ± 2.5	111.3 ± 0.6
Zn^2+^	85.3 ± 2.1	53.9 ± 1.4
Ca^2+^	125.8 ± 1.1	166.4 ± 1.9
Co^2+^	101.9 ± 0.3	72.3 ± 1.3
Cu^2+^	56.8 ± 4.5	24.3 ± 2.0
Fe^2+^	42.0 ± 3.1	25.6 ± 1.4
Fe^3+^	18.5 ± 2.0	22.9 ± 1.0
Mg^2+^	89.3 ± 2.4	83.6 ± 0.1
Mn^2+^	102.3 ± 3.9	112.5 ± 2.5
Pb^2+^	45.4 ± 2.1	74.3 ± 4.8
Sb^3+^	10.1 ± 0.1	7.4 ± 0.2
Al^3+^	67.6 ± 1.5	47.3 ± 1.7
Bi^3+^	10.3 ± 1.1	5.5 ± 0.8

Enzyme samples were incubated with various metal ions (1 and 10 mM) for 30 min, and the relative activity was assessed using the standard assay protocol. Results are presented as means ± standard deviation (*n* = 3).

**Table 3.** Effects of various inhibitors and detergents on the activity of lipase.

	Relative activity (%)		
	1 mM (0.1%, *v/v*)	10 mM (1%, *v/v*)	
Control	100.0 ± 1.5	100.0 ± 1.0
Inhibitors		
DTT	85.3 ± 2.1	71.5 ± 1.7
EDTA	66.5 ± 2.4	32.9 ± 3.3
H_2_O_2_	88.9 ± 1.1	76.8 ± 1.4
Detergents		
CTAB	107.8 ± 2.0	91.5 ± 0.6
SDS	94.6 ± 0.2	89.2 ± 3.1
Sodium cholate	86.6 ± 2.6	80.3 ± 4.4
Tween-20	104.3 ± 1.3	94.5 ± 1.0
Tween-60	97.6 ± 2.2	92.1 ± 1.9
Tween-80	120.3 ± 0.8	105.7 ± 3.4
Triton X-100	104.9 ± 1.7	96.2 ± 1.9

Inhibitors and detergents (1 and 10 mM, 0.1 and 1% *v/v*) were added in the reaction mixture and assayed using standard assay protocol. Results are presented as means ± standard deviation (*n* = 3).

#### 2.9.4. Determination of reaction kinetics and substrate specificity

The maximum specific activity (*V*_max_) and Michaelis-Menten constant (*K*_m_) in reaction kinetics of SCNL was determined by Lineweaver-Burk plots using Origin software. The specific activity of SCNL was determined with different concentrations of *p*-NPP (0.1–5.0 mM) under optimum conditions.

The specificity of substrate with varying chain length and different saturation was investigated by the colorimetric and titrimetric assay. Furthermore, the specific activity and relative activity of SCNL towards the *p*-NP fatty acid esters (C_2_–C_18_), triglycerides (C_2_–C_18_) and nature oil were measured under different standard assay conditions according to a previous method^14^, with minor modifications.

### 2.10. Statistical analysis

Results were expressed as mean ± standard deviation (SD). One-way analysis of variance (ANOVA), followed by Tukey's *post hoc* test, was used to determine statistically significant differences (*p* < 0.05) between the treatment groups and the control by using SPSS 19.0 statistical software (IBM Corp., Armonk, NY, USA).

## 3. Results and discussion

### 3.1. Isolation and identification of the lipolytic strain

It was reported that the lipases were generally produced in the presence of lipid sources, such as olive oil^15^. A total of 36 bacterial isolates were identified from the sewerage samples collected at Nanchang City, Jiangxi Province, China. In the preliminary screening, only 22 isolates showed clear halo on the bromocresol purple plates after 24-h cultivation at 37 °C, among them 13 isolates also produced a zone of clearance surrounding each colony after 2-day incubation on tributyrin agar plates. However, the tributyrin was not only sensitive to hydrolysis by lipases, but also by esterases. Rescreening of the 13 isolates found that only 6 of them showed obvious effect of lipase production on Rhodamine B agar plate. NCU S6 was found to be the best lipolytic strain that showed the broadest orange fluorescence halos around each colony through UV irradiation.

**Figure 1.**
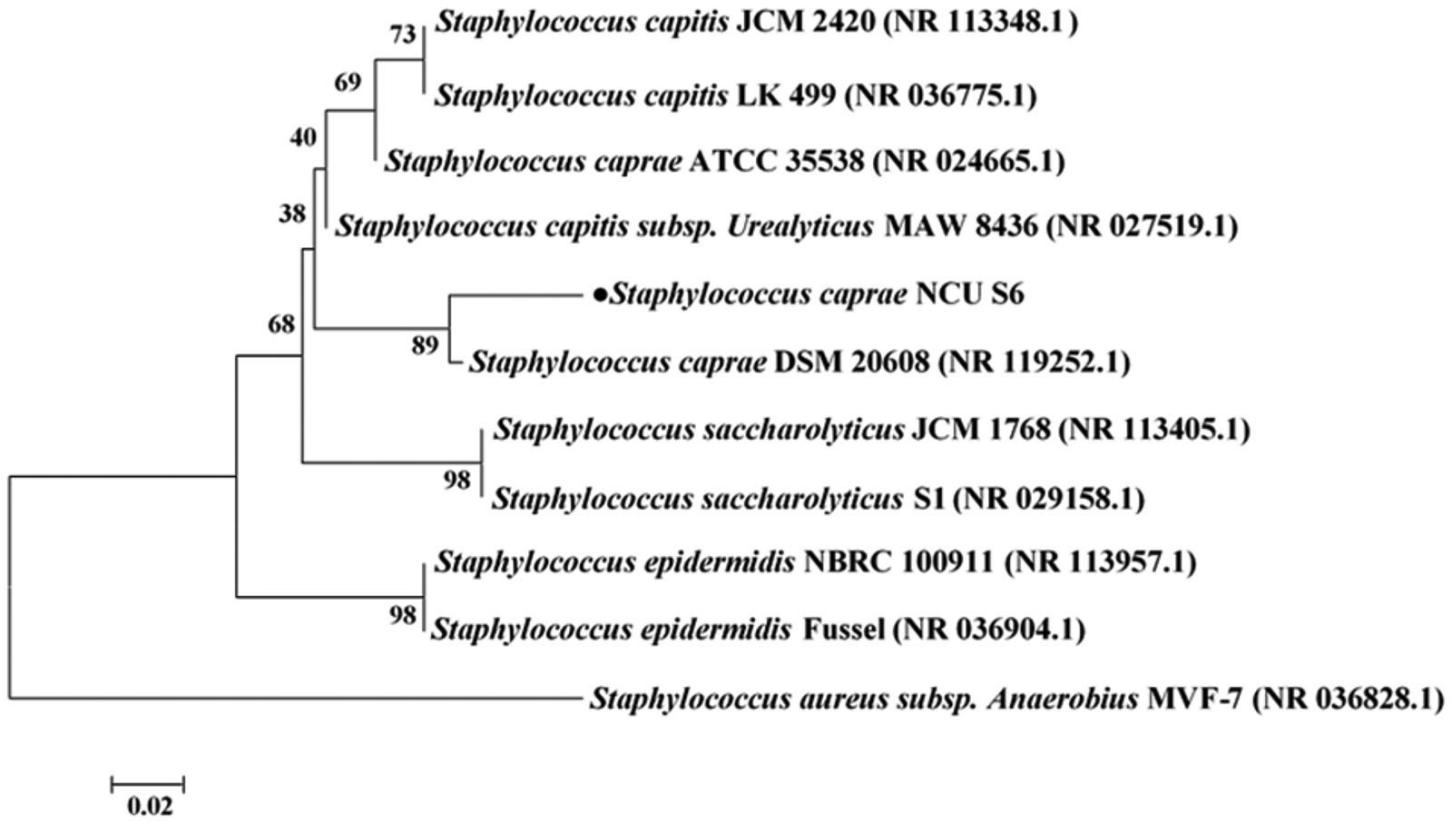
**A p**hylogenetic tree displaying the relationship between strain NCU S6 and other *Staphylococcus* species based on partial 16S rDNA sequences of the strains of the genus. The tree was constructed using a neighbour joining algorithm in MEGA 7.0 software and the accession numbers are shown in parenthesis.

**Figure 2.**
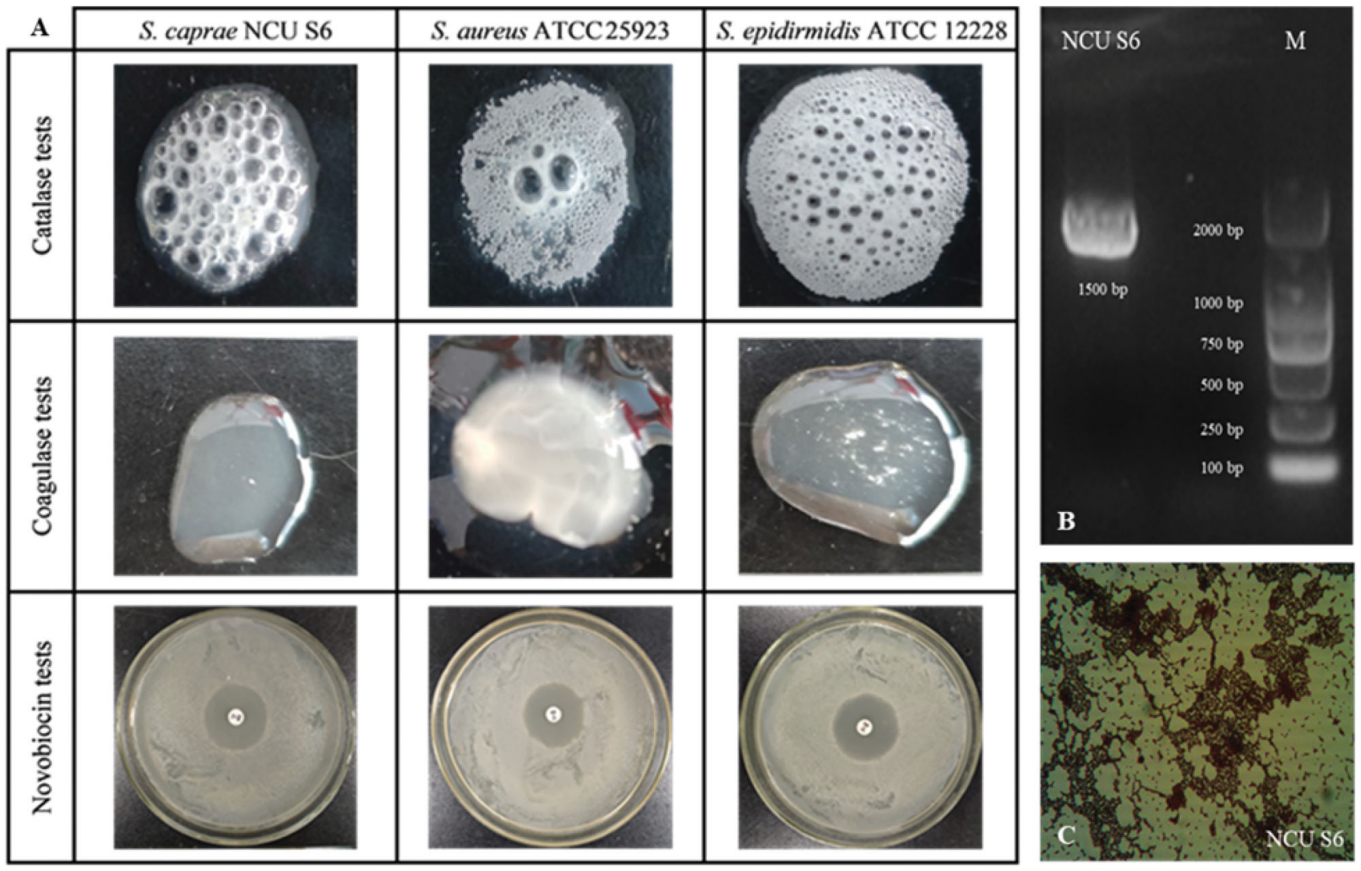
(A) Results of coagulase tube test, novobiocin (5 μg/piece) sensitivity test, and catalase production test. (B) The result of 16S rDNA agarose gel electrophoresis analysis. (C) The image of Gram-staining result of *S. caprae* NCU S6.

The 16S rDNA V1-V9 region amplified by PCR was sequenced, and subjected to the homology searches using NCBI BLAST analysis. The results revealed a 99% identity with different species belonging to *Staphylococcus* genus, and the strain was named *Staphylococcus caprae* NCU S6. The phylogenetic analysis (Figure 1) showed that the strain NCU S6 was the most closely (89%) related to *S. caprae* strain DSM 20608. The gram staining of NCU S6 showed gram positive. Biochemical approaches, including catalase, coagulase and novobiocin tests, were used to identify the Staphylococcus genus. As shown in Figure 2(A), all three *Staphylococcus* strains instantly produced lots of bubbles, indicating catalase positive. *S. caprae* NCU S6 was found to be coagulase-negative and sensitive to novobiocin (radius of inhibition zone ≤10 mm). The reference strains, *S. aureus* ATCC 25923 (negative control) and *S. epidermidis* ATCC 12228 (positive control) were coagulase-positive and coagulase-negative respectively, and both exhibited obvious resistance on novobiocin agar plate (radius of inhibition zones ≥18 mm). The results have shown that NCU S6 strain belonged to *S. caprae* species which were not pathogens.

### 3.2. Growth and lipase activity characteristics of NCU S6

**Figure 3.**
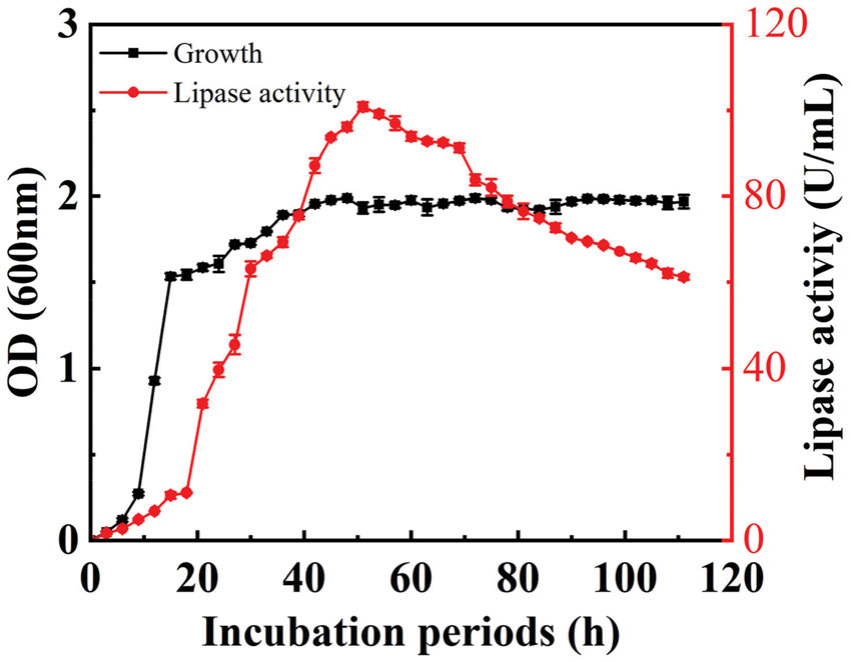
The time courses of cell growth (▲) of *S. caprae* NCU S6 and lipase production (■). The culture was carried out at 37 °C and 200 rpm for 111 h and cell growth was monitored by measuring the absorbance at 600 nm every 3 h.

NCU S6 strain was cultivated in growth medium A, and the cell metabolism was strongly linked to lipase activity. With the increase of incubation period, enzyme production was firstly increased and then decreased. As shown in Figure 3, dynamic growth condition and lipase activity of the cells in the fermentation medium was reflected by absorbance value. The logarithmic growth phase of NCU S6 started quickly after culturing in fermentation medium for 3 h and lasted for approximately 21 h. A slow growth from 24 to 39 h was observed, and the strain subsequently entered a stationary phase from 42 to 111 h. Figure 3 also showed the dynamic changes of lipase activity, with an increase from the beginning of inoculation to 51 h. The growth rate declined from 51 to 111 h, which may result from the depletion of nutrients in the fermentation medium and a low metabolism level under aging phase of growth^16^. Obviously, the peak (51 h) of lipase activity (101 U/mL) in the culture period was the optimal enzyme production time.

### 3.3. Purification of *S. caprae* lipase

The extracellular lipase from *S. caprae* NCU S6 (SCNL) was purified by three purification steps, ASP, GFC and IEC. As shown in Table 4, the activity (502.76 U/mg) of the purified SCNL was increased by 332.95-fold, with approximately 6.13% recovery of overall yield. Among the three purification steps, GFC and IEC were found to be critical for obtaining highly purified lipase^17^.

**Table 4.** Purification profile of lipase from *S. caprae*.

Purification steps	Total activity (U^a^)	Total protein^b^ (mg)	Protein yield (%)	Activity (%)	Specific activity (U/mg protein)	Purification (fold)
Crude extract	6154.11	4080.00	100.00	100.00	1.51	1.00
(NH_4_)_2_SO_4_ precipitation (80%)	3692.47	652.00	39.33	54.67	5.66	3.75
Ultrafiltration	1891.06	24.00	15.29	30.73	78.79	52.18
Gel filtration chromatography	1250.09	6.45	5.31	20.31	193.81	128.35
Ion-exchange chromatography	626.67	1.85	3.67	10.18	338.74	224.33
Ultrafiltration	377.07	0.75	1.77	6.13	502.76	332.95

^a^One unit corresponds to 1 μmol of fatty acid released per min using *p*-NPP as substrate under standard conditions.

^b^Protein concentration was determined by Bradford method.

**Figure 4.**
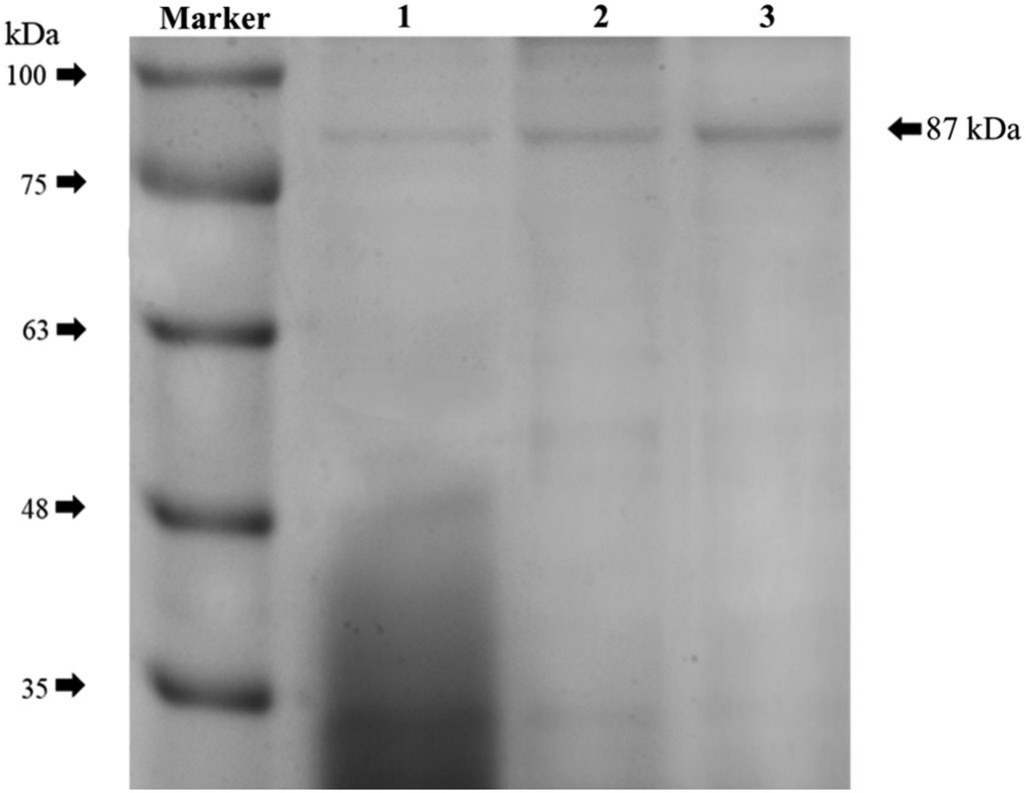
SDS–PAGE electrophoretogram of the lipase from different purification stages. Lane 1: crude enzyme, Lane 2: purified enzyme from ion-exchange chromatography, Lane 3: final purified enzyme from gel filtration chromatography.

SDS-PAGE analysis was used to determine the purity of SCNL obtained after purification. As shown in Figure 4, the purified lipase showed a single band, with a molecular weight of approximately 87 kDa.

### 3.4. Biochemical characteristics

#### 3.4.1. Optimal temperature and thermostability

**Figure 5.**
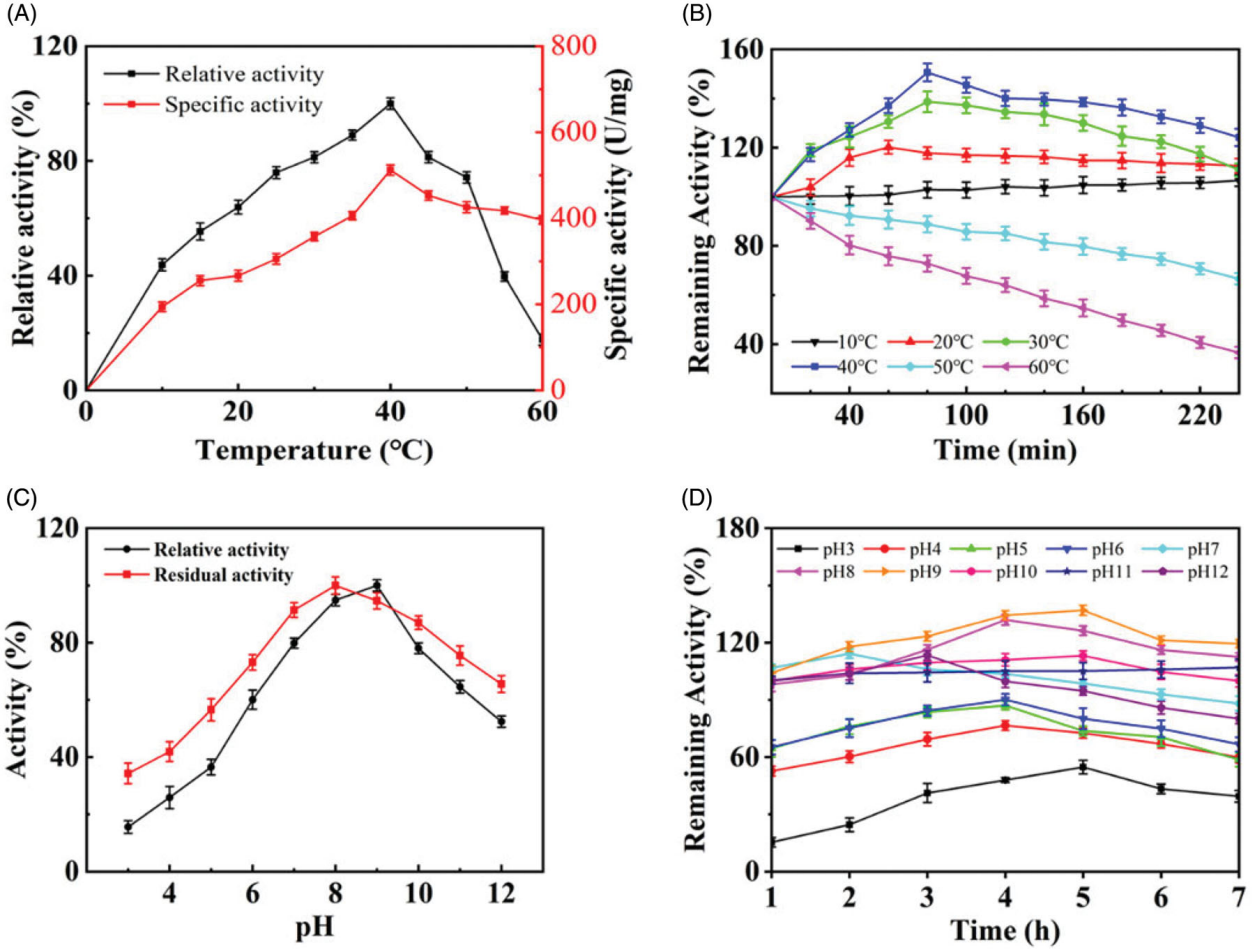
Effect of temperature (A) and temperature-time (B), pH (C) and pH-time (D) on activity and stability of the lipase.

Temperature is one of the most important factors affecting the reaction rate of enzyme catalysis. As shown in Figure 5(A), the lipase was active in the wide range of temperatures from 10 to 60 °C. The maximum hydrolytic activity was found at 40 °C, with the relative activity and specific activity of 100% and 503 U/mg respectively. The optimal reaction temperature was lower than the lipase from *Aureobasidium pullulans* (55 °C)^18^. Activity analyses of the lipase at temperatures from 10 to 60 °C for 240 min showed that the remaining activity at 40 °C was at the highest level (Figure 5(B)). Only the extremely high temperatures, such as 50 or 60 °C, significantly inhibited the lipase activity, and prolonged incubation may rapidly inactivate the lipase. The highest remaining activity was at 40 °C for 80 min, and the lipase activity decreased with an increase of culture period. The decrease may be because the molecular structure of the enzyme was irreversibly changed, which may have altered the configuration of the active site, thereby decreasing interaction of the lipase with substrates^19^.

#### 3.4.2. Optimal pH and tolerance of lipase

The effect of pH on lipase activity and stability was determined by pre-incubating the enzyme in the pH values from 3.0 to 12.0 for 30 min at 40 °C. As shown in Figure 5(C), the lipase was active (≥50%) from pH 5.5 to 12.0, with the highest relative activity and residual activity at pH 9.0 and 8.0, respectively. The enzyme was similar to the lipase from *Serratia marcescens* N3, which showed maximum activity at pH 8.0^20^. Investigation of pH tolerance of the enzyme (3.0–12.0, Figure 5(D)) showed that the enzyme reached the highest remaining activity at pH 9.0 after 5 h. The lipase possessed a strong tolerance to alkaline environment, and the remaining activity was greater than 100% from pH 8.0 to 11.0. The stability of SCNL under alkaline conditions was also observed in *S. aureus* and *S. epidermis*^21^. The enzyme activities at pH 3.0–6.0 were lower than those at the neutral pH, with 60% of its initial activity being lost at pH 3.0. The results suggested that the lipase was suitable for applications under alkaline conditions.

#### 3.4.3. Effects of organic solvents on the stability of the lipase

Lipases with tolerance to organic solvents have received a great attention in recent years, as the enzymes could be used as desirable catalysts in various biotechnological fields, such as biofuels production, synthesis of catalysts and the optical resolution of chiral compounds^22^. In the current study, the lipase showed a strong stability in several organic solvents at 10% (*v/v*) for 1 day, and its activity slightly decreased in acetone, ethyl acetate, diethyl ether, ethylene glycol and acetonitrile after 28-day incubation. These results were consistent with a previous study showing that DMSO at 10% (*v/v*) improved the stability of the enzyme by dissolving the protein to some extent^23^. Only some organic solvents enhanced the residual activity of the enzyme at 10–50% for 1 or 28 days, and the effects of isooctane and glycerol on the enzyme activity were similar to the lipases from *Pseudomonas aeruginosa* PseA^24^. Besides, even after preincubation in 50% (*v/v*) acetone for 28 days the residual activity of the lipase was still 50% of its initial activity, indicating that it could be used as a functional catalyst for applications in the synthesis of esters.

#### 3.4.4. Effects of metal ions on the stability of the lipase

The tolerance of an enzyme to various metal ions is crucial not only for investigating its action mechanism, but also for its certain industrial applications. Several metal ions were reported to be involved in the lipase-catalyzed hydrolysis of oil by inducing the fatty acids to form their respective metal salts in the oil–water interface and allowing lipase to act freely on oil molecules^25^. The effects of monovalent, divalent and trivalent metal cations on lipase activity were analysed at 1 and 10 mM. As shown in Table 2, the lipase activity was significantly improved in the presence of Na^+^, Ba^2+^, Ca^2+^ and Mn^2+^. Especially, its relative activity in the presence of 10 mM Ca^2+^ was 1.66 folds higher than the control. Ca^2+^ was reported to play an important role in conformational stability of the enzyme^26^. Among the tested metal ions, Sn^2+^, Zn^2+^, Cu^2+^, Fe^2+^, Fe^3+^, Al^3+^, Sb^3+^ and Bi^3+^ significantly inhibited the relative activity.

#### 3.4.5. Effects of inhibitors and detergents on the stability of the lipase

Commercial inhibitors and detergents were important surfactants for the preparation of emulsions in their practical applications. As shown in Table 3, ethylenediaminetetraacetic acid (EDTA, 1 and 10 mM) exhibited significant effects on its relative activity (66.5 and 32.9%), indicating that SCNL was not only a metalloenzyme, but also a metal-activated enzyme. The inhibitors, H_2_O_2_ and dithiothreitol (DTT) were reported to reduce the disulphide bonds of an enzyme, and prevent the formation of inter- or intramolecular disulphide bonds between cysteine residues of a lipase^27^, but the relative activities of the lipase in the presence of these two inhibitors at 1 and 10 mM still maintained more than 70%.

The stability of lipases in the presence of detergents is the premise of being good washing additives in commercial detergents. In addition, surfactants can stimulate lipases in molecular bioimprinting, and play important roles in multifunctionality and various properties of enzymes^28^. Interestingly, cationic detergent cetyltrimethylammonium bromide (CTAB) and anionic detergent (sodium dodecyl sulphate (SDS) slightly decreased the relative activity of SCNL, which disagreed with the lipase from *Burkholderia ubonensis* SL-4^29^. As shown in Table 3, the non-ionic detergents slightly improved lipase activity. The non-ionic detergents did not weaken the hydrophobic interactions within the protein and damage the lipase^30^. In addition, bile salts, such as sodium cholate, an universal commercial biosurfactant as a reducing agent in aqueous solutions slightly restrained the activity at 1 or 10 mM, as the lipase still retained above 80% of initial activity. These findings were similar to a previous study showing that the bile salts did not restrain the activity of *S. xylosus* lipase even at higher concentrations^31^. Therefore, SCNL may be a novel additive for the industrial applications of various multifunctional commercial detergents.

#### 3.4.6. *K_m_*, *V*_max_ and substrate specificity

The *K*_m_ and *V*_max_ values of SCNL were determined to be 0.695 mM and 262.66 s^−1 ^mM^−1^ respectively, when the substrate was *p*-NPP. The *K*m value was slightly higher than those of KM12 lipase (0.53 mM)^32^ and the lipase from *B. licheniformis* strain SCD11501 (0.43 mM)^33^. The *V*max value of SCNL was higher than those of KM12 lipase (171.2 s^−1 ^mM^−1^)^32^ and SML lipase (149.03 s^−1 ^mM^−1^)^34^. The results indicated the strong attraction of SCNL for the main substrate, and its high catalytic efficiency, and SCNL may be used as a novel lipase in pharmaceutical and biotech fields due to its notable economic and ecological benefits^35^.

The SCNL can hydrolyse *p*-NP esters with different acyl chain length (Table 5), with the highest specific activity of 616.54 U/mg towards *p*NPS (18 carbons). The *p*-NP esters from C_2_ to C_16_ were hydrolysed moderately (45.46–54.54%) by SCNL apart from *p*NPB (4 carbons), indicating its preference for C_4_ with a higher specific activity of 479.91 U/mg. The lipase was similar to the lipases from *M. cinnamomea*^36^ and *B. licheniformis* DSM 12369^24^, possessed a strong hydrolysis efficiency for medium and long carbon chain length substrates, and had mild specificity towards short-chain *p*-NP esters. Among the triglycerides, SCNL showed a preference for short-chain and medium-chain fatty acids from C_2_ to C_12_, with the maximum activity (196.00 U/mg) in tributyrin (C_4_). The results indicated a lower hydrolytic activity of the enzyme for triglycerides with more than twelve carbons atoms (C_14_–C_18_), and that SCNL was a novel lipase different from esterases that only hydrolyse short-chain fatty acids.

**Table 5.** Substrate specificity of SCNL.

Substrate	Specific activity (U/mg)	Relative activity (%)
*p*NP ester^a^		
* p*NPA (*p*-nitrophenyl acetate) (C_2_)	301.20 ± 7.96	48.85
* p*NPB (*p*-nitrophenyl butyrate) (C_4_)	479.91 ± 1.79	77.84
* p*NPH (*p*-nitrophenyl hexanoate) (C_6_)	281.98 ± 3.08	45.74
* p*NPC (*p*-nitrophenyl caprylate) (C_8_)	280.28 ± 6.21	45.46
* p*NPD (*p*-nitrophenyl decanoate) (C_10_)	336.23 ± 6.23	54.54
* p*NPL (*p*-nitrophenyl laurate) (C_12_)	312.72 ± 1.92	50.72
* p*NPM (*p*-nitrophenyl myristate) (C_14_)	310.45 ± 2.50	50.35
* p*NPP (*p*-nitrophenyl palmitate) (C_16_)	334.72 ± 1.14	54.29
* p*NPS (*p*-nitrophenyl stearate) (C_18_)	616.54 ± 5.96	100.00^c^
Triglyceride^b^		
Triacetin (C_2_)	71.05 ± 6.00	36.25
Tributyrin (C_4_)	196.00 ± 5.29	100.00^c^
Tricaproin (C_6_)	60.43 ± 6.11	30.83
Tricaprylin (C_8_)	49.00 ± 4.00	25.00
Tricaprin (C_10_)	24.01 ± 3.42	12.25
Trilaurin (C_12_)	29.40 ± 4.00	15.00
Trimyristin (C_14_)	28.58 ± 3.06	14.58
Tripalmitin (C_16_)	24.50 ± 5.29	12.50
Tristearin (C_18_)	22.87 ± 4.62	11.67
Natural oils^b^		
Olive oil	184.80 ± 3.59	84.00
Soybean oil	155.47 ± 4.15	70.67
Rice bran oil	136.69 ± 3.96	62.13
Flaxseed oil	153.41 ± 2.90	69.73
Perilla oil	142.56 ± 1.90	64.80
Canola oil	220.00 ± 7.19	100.00^c^
Corn oil	150.48 ± 0.72	68.40

^a^Unit definition: 1 unit of activity is the amount of enzyme necessary to release 1 μmol of *p*-NP per minute at 37 °C and pH 7.0 for SCNL.

^b^Unit definition: 1 unit of activity is the amount of enzyme necessary to release 1 μmol of free fatty acids per minute at 37 °C and pH 7.0 for SCNL.

^c^The highest activity of SCNL was taken as 100% by using different substrates.

Among the tested natural oils, the canola oil was hydrolysed to a higher degree by SCNL at a high specific activity (220.00 U/mg). The canola oil contains less than 2% erucic acid and less than 30 mM glucosinolates, and has a relatively low level of saturated fat (≤7%) and a high content of polyunsaturated fats, such as linoleic acid and α-linolenic acid (formally called 9,12,15-all-cis-octadecatrienoic acid). α-linolenic acid is an unsaturated omega-3 fatty acid available in many plant oils. The activity of SCNL was strong for natural oils with medium-chain to long-chain fatty acids [C_6:0_–C_16:0_]. Among them, both olive oil (184.80 U/mg) and rice bran oil (176.69 U/mg) contain long unsaturated fatty acids, such as oleic acid and linoleic acid. These results were in accordance with a previous report^14^, indicating its preference to medium-chain fatty acids and long unsaturated fatty acids.

## 4. Conclusions

A novel and multifunctional lipase, SCNL was purified from a newly isolated *S. caprae* NCU S6. The molecular weight of the lipase was determined to be approximately 87 kDa through gel electrophoresis analysis. The lipase exhibited high temperature resistance and great pH stability. Furthermore, SCNL was highly active in the presence of a variety of industrial organic solvents and metal ions. The lipase also exhibited strong tolerance to widely used enzyme inhibitors and commercial detergents. Our results suggested that SCNL could be an excellent enzyme candidate for biotransformation in the food and pharmaceutical industries. Further studies would be focussed on its region-specificity and structural analysis, and its potential applications in biodiesel production and detergent industries.

## References

[1] Javed S, Azeem F, Hussain S, et al. Bacterial lipases: a review on purification and characterization. Prog Biophys Mol Biol 2018;132:23–34. 28774751 10.1016/j.pbiomolbio.2017.07.014

[2] Sarmah N, Revathi D, Sheelu G, et al. Recent advances on sources and industrial applications of lipases. Biotechnol Prog 2018;34:5–28. 29086509 10.1002/btpr.2581

[3] Sharma R, Soni SK, Vohra RM, et al. Purification and characterisation of a thermostable alkaline lipase from a new thermophilic *Bacillus* sp. Rsj-1. Process Biochemistry 2002;37:1075–84.

[4] Lumor SE, Akoh CC. Esterification and hydrolytic activities of candida rugosa lipase isoform 1 (lip1) immobilized on celite 545, duolite a7, and sephadex g-25. J Agric Food Chem 2008;56:10396–8. 18850711 10.1021/jf802136d

[5] Ji X, Chen G, Zhang Q, et al. Purification and characterization of an extracellular cold-adapted alkaline lipase produced by psychrotrophic bacterium *Yersinia enterocolitica* strain KM1. J Basic Microbiol 2015;55:718–28. 25677080 10.1002/jobm.201400730

[6] Cadieux B, Vijayakumaran V, Bernards MA, et al. Role of lipase from community-associated methicillin-resistant staphylococcus aureus strain usa300 in hydrolyzing triglycerides into growth-inhibitory free fatty acids. J Bacteriol 2014;196:4044–56. 25225262 10.1128/JB.02044-14 PMC4248881

[7] Samad MYA, Razak CNA, Salleh AB, et al. A plate assay for primary screening of lipase activity. J Microbiol Meth 1989;9:51–6.

[8] Kouker G, Jaeger KE. Specific and sensitive plate assay for bacterial lipases. Appl Environ Microbiol 1987;53:211–3. 3103532 10.1128/aem.53.1.211-213.1987 PMC203632

[9] Ferreira AM, Bonesso MF, Mondelli AL, da Cunha M. d L R d S. Identification of *Staphylococcus saprophyticus* isolated from patients with urinary tract infection using a simple set of biochemical tests correlating with 16s-23s interspace region molecular weight patterns. J Microbiol Methods 2012;91:406–11. 23041266 10.1016/j.mimet.2012.09.024

[10] Gricajeva A, Bendikienė V, Kalėdienė L. Lipase of bacillus stratosphericus l1: Cloning, expression and characterization. Int J Biol Macromol 2016;92:96–104. 27392776 10.1016/j.ijbiomac.2016.07.015

[11] Gricajeva A, Bikutė I, Kalėdienė L. Atypical organic-solvent tolerant bacterial hormone sensitive lipase-like homologue estag1 from *Staphylococcus saprophyticus* ag1: Synthesis and characterization. Int J Biol Macromol 2019;130:253–65. 30797006 10.1016/j.ijbiomac.2019.02.110

[12] Bradford MM. A rapid and sensitive method for the quantitation of microgram quantities of protein utilizing the principle of protein-dye binding. Anal Biochem 1976;72:248–54. 942051 10.1016/0003-2697(76)90527-3

[13] Laemmli UK. Cleavage of structural proteins during the assembly of the head of bacteriophage T4. Nature 1970;227:680–5. 5432063 10.1038/227680a0

[14] Chen CC, Gao GJ, Kao AL, et al. Two novel lipases purified from rice bran displaying lipolytic and esterification activities. Int J Biol Macromol 2019;139:298–306. 31386870 10.1016/j.ijbiomac.2019.08.026

[15] Boekema BKHL, Beselin A, Breuer M, et al. Hexadecane and tween 80 stimulate lipase production in *Burkholderia glumae* by different mechanisms. Appl Environ Microbiol 2007;73:3838–44. 17468265 10.1128/AEM.00097-07 PMC1932709

[16] Rmili F, Achouri N, Smichi N, et al. Purification and biochemical characterization of an organic solvent-tolerant and detergent-stable lipase from *Staphylococcus capitis*. Biotechnol Prog 2019;35:e2833. 31050178 10.1002/btpr.2833

[17] Castro-Ochoa LD, Rodríguez-Gómez C, Valerio-Alfaro G, Ros RO. Screening, purification and characterization of the thermoalkalophilic lipase produced by *Bacillus thermoleovorans* ccr11. Enzyme Microbial Technology 2005;37:648–54.

[18] Li Y, Liu TJ, Zhao MJ, et al. Screening, purification, and characterization of an extracellular lipase from *Aureobasidium pullulans* isolated from stuffed buns steamers. J Zhejiang Univ Sci B 2019;20:332–42. 30932378 10.1631/jzus.B1800213 PMC6454315

[19] Das A, Shivakumar S, Bhattacharya S, et al. Purification and characterization of a surfactant-compatible lipase from aspergillus tamarii jgif06 exhibiting energy-efficient removal of oil stains from polycotton fabric. Biotechnol 2016;6:131. 10.1007/s13205-016-0449-z PMC4909032 28330188

[20] Neihaya HZ, Saeed SE, Baho S. Production, purification and characterization of extra cellular lipase from *Serratia marcescens* and its potential activity for hydrolysis of edible oils. Al Nahrain Univ 2012;15:94–102.

[21] Xie W, Khosasih V, Suwanto A, Kim HK. Characterization of lipases from *Staphylococcus aureus* and *Staphylococcus epidermidis* isolated from human facial sebaceous skin. J Microbiol Biotechnol 2012;22:84–91. 22297223 10.4014/jmb.1107.07060

[22] Dandavate V, Jinjala J, Keharia H, Madamwar D. Production, partial purification and characterization of organic solvent tolerant lipase from *Burkholderia multivorans* v2 and its application for ester synthesis. Bioresour Technol 2009;100:3374–81. 19285387 10.1016/j.biortech.2009.02.011

[23] Zhao LL, Xu J-H, Zhao J, et al. Biochemical properties and potential applications of an organic solvent-tolerant lipase isolated from *Serratia marcescens* ecu1010. Process Biochem 2008;43:626–33.

[24] Gaur R, Gupta A, Khare SK. Purification and characterization of lipase from solvent tolerant *Pseudomonas aeruginosa* psea. Process Biochemy 2008;43:1040–6.

[25] Sharon C, Nakazato M, Ogawa HI, Kato Y. Lipase-induced hydrolysis of castor oil: effect of various metals. J Indus Microbiol Biotechnol 1998;21:292–5.

[26] Invernizzi G, Papaleo E, Grandori R, et al. Relevance of metal ions for lipase stability: structural rearrangements induced in the *Burkholderia glumae* lipase by calcium depletion. J Struct Biol 2009;168:562–70. 19635571 10.1016/j.jsb.2009.07.021

[27] Phukon LC, Chourasia R, Kumari M, et al. Production and characterisation of lipase for application in detergent industry from a novel *Pseudomonas helmanticensis* hs6. Bioresource Technology 2020;309:123352. 32299046 10.1016/j.biortech.2020.123352

[28] Mukherjee J, Gupta MN. Molecular bioimprinting of lipases with surfactants and its functional consequences in low water media. Int J Biol Macromol 2015;81:544–51. 26306412 10.1016/j.ijbiomac.2015.08.033

[29] Yang W, He Y, Xu L, et al. A new extracellular thermo-solvent-stable lipase from *Burkholderia ubonensis* sl-4: identification, characterization and application for biodiesel production. J Mol Catal B 2016;126:76–89.

[30] Kukreja V, Bera MB. Lipase from pseudomonas aeruginosa mtcc 2488: partial purification, characterization and calcium dependent thermostability. Ind J Biotechnol 2005;4:222–6.

[31] Bouaziz A, Horchani H, Salem NB, et al. Expression, purification of a novel alkaline *Staphylococcus xylosus* lipase acting at high temperature. Biochem Eng J 2011;54:93–102.

[32] Malekabadi S, Badoei-Dalfard A, Karami Z. Biochemical characterization of a novel cold-active, halophilic and organic solvent-tolerant lipase from b. Licheniformis km12 with potential application for biodiesel production. Int J Biol Macromol 2018;109:389–98. 29258898 10.1016/j.ijbiomac.2017.11.173

[33] Sharma S, Kanwar SS. Purification and bio-chemical characterization of a solvent-tolerant and highly thermostable lipase of *Bacillus licheniformis* strain scd11501. Proc Natl Acad Sci India Sect B Biol Sci 2017;87:411–9.

[34] Li M, Yang LR, Xu G, Wu JP. Screening, purification and characterization of a novel cold-active and organic solvent-tolerant lipase from *Stenotrophomonas maltophilia* cgmcc 4254. Bioresour Technol 2013;148:114–20. 24050922 10.1016/j.biortech.2013.08.101

[35] Sarkar P, Yamasaki S, Basak S, et al. Purification and characterization of a new alkali-thermostable lipase from *Staphylococcus aureus* isolated from Arachis hypogaea rhizosphere. Process Biochem 2012;47:858–66.

[36] Duan X, Xiang M, Wang L, et al. Biochemical characterization of a novel lipase from *Malbranchea cinnamomea* suitable for production of lipolyzed milkfat flavor and biodegradation of phthalate esters. Food Chem 2019;297:124925. 31253266 10.1016/j.foodchem.2019.05.199

